# Medicine and generative artificial intelligence

**DOI:** 10.47626/1679-4435-2024-221

**Published:** 2024-08-05

**Authors:** Chao Lung Wen

**Affiliations:** Associate professor at Universidade de São Paulo (full professor) and head of Disciplina de Telemedicina, Departamento de Patologia at Faculdade de Medicina da Universidade de São Paulo. President of Associação Brasileira de Telemedicina e Telessaúde (ABTms, Brazilian Association of Telemedicine and Telehealth). Coordinator at Núcleo de Desenvolvimento Organizacional da Saúde Digital, Hospital das Clínicas da Faculdade de Medicina da Universidade de São Paulo

At first glance, artificial intelligence (AI) is considered a new technology (in the last 10 years), but in fact it emerged from researchers aiming to create flexible machines capable of performing tasks that require human intelligence. John McCarthy, Marvin Minsky, and Allen Newell coined the term and presented it at a conference in Dartmouth in 1956, an event that is now considered the starting point for AI.

AI is a branch of Computer Science that is defined as simulating the intelligent behavior of machines. Originally, AI consisted of developing computer programs capable of performing specific tasks, like playing chess or solving complex mathematical problems. Applications already existed in the medical field in the 1970s were used with limitations due to the inexistence of structured information in databases and even the unavailability of powerful processors like those used today. AI began to evolve more rapidly from the beginning of the twenty-first century as a result of improvements in software algorithms, computer processing capacity, and the availability of large volumes of data (cloud storage). AI software can be classified in various ways, as described below:

1. Artificial unintelligence (AU)-also called weak, narrow, or limited artificial intelligence-is nearly all of currently used AIs. They have been developed to perform specific tasks. They can store large amounts of data and handle complex tasks, but they are always focused on the objective for which they were programmed and cannot perform other tasks independently. Examples are Waze/Google Maps, AI used in radiology, dermatology, among others.
2. Generative artificial intelligence (GAI) are flexible AI systems designed to deal with a wide range of tasks and problems in a similar way to human beings. Possibly, some model will be launched in the next 5 years.
3. Artificial superintelligence (ASI) are AI systems that could surpass individual human intelligence and master a wide range of tasks or deal with many types of problems. It is accepted that this still theoretical AI model would only become feasible after the emergence of quantum computers.

## GENERATIVE ARTIFICIAL INTELLIGENCE (GAI)

OpenAI ChatGPT 3.5 was launched in November 2022, and the category of AI with an algorithm core that allows it to create or generate new data, content, or information independently and unprecedentedly from results obtained from an internal search of a pretrained AI has become increasingly popular. This AI has the ability to create something new, often imitating patterns and styles learned during training. It is an AI system capable of interacting in natural conversation to answer questions sent in text form and replying also as text. The ability to generate content autonomously makes GAI a versatile tool for many creative and practical applications. However, it is worth recalling that although GAI has shown impressive advances, it has challenges, such as the potential to generate false or biased information, due to insufficient training or having used information obtained from the open Internet, with neither the quality of scientific journal databases, peer-reviewed data, nor adequate methods, generating the risk of inefficient training. It is therefore important to stress that GAIs are evolving and that the results often need to be checked against specific literature bases for validation purposes. The responsibility for deciding whether to accept the AI response as true or valid lies with the GAI user or GAI professional.

When using an AI platform, assess its degree of reliability is necessary. A number of criteria can be deemed as needed:

- Check the database used for training (general or indexed scientific data);
- Frequency of updates;
- Data inclusion and exclusion criteria;
- Existence of a regular results evaluation team (audit); and
- Periodically checking the level of accuracy of responses.

While GAIs are useful for numerous purposes, they need to be used appropriately and judiciously, especially when applied for professional and specialized purposes. One example is ChatGPT. It was not developed specifically for the health sector, nor was it trained using scientific databases. As a result, their responses may contain error biases. The company OpenAI is launching a version of ChatGPT Enterprise, which aims to free up the algorithm to be trained with company-specific databases, resulting in subject-specific AI, reducing the occurrence of errors.

Good results from a GAI system also depend on how the questions are asked (question quality). It is difficult to get good answers unless you know how to ask good questions with sufficient specificity.

In the professional setting with AI in everyday use, physicians and health care personnel have to focus on the following skills:

- Being ethical;
- Having curiosity;
- Having observation skills;
- Ability to ask good questions;
- Ability to search for information;
- Ability to compare answers and interpret them according to the context;
- Ability to make decisions;
- Good communication skills and empathy;
- Adaptability to new situations; and
- Ability to use new technologies.

GAI racing has increased greatly since ChatGPT was launched, and companies such as Microsoft (CoPilot), Google (Gemini), Meta AI (Facebook), and Amazon, among others, have launched their GAI platforms. As a result of various issues related to the use of AI, there are ongoing discussions, and the World Health Organization (WHO) and Conselho Federal de Medicina (CFM, Brazilian Federal Council of Medicine) have published both Ethics and governance of artificial intelligence for health^1^ and ordinance No. SEI-39/2024, of March 2024, which established the Grupo de trabalho de regulamentação da inteligência artificial na medicina (Artificial intelligence regulation workplan to guide use of AI in Medical Sciences).^2^
